# Transient Humoral Protection against H5N1 Challenge after Seasonal Influenza Vaccination of Humans

**DOI:** 10.1371/journal.pone.0103550

**Published:** 2014-07-30

**Authors:** Ramon Roozendaal, Jeroen Tolboom, Anna Roos, Sarra Riahi, Jessica Theeuwsen, Miriam V. Bujny, Vincent Klaren, Hans J. W. M. Korse, Liesbeth Dekking, Arijan Grootenhuis, Gerrit Jan Weverling, Wouter Koudstaal, Jaap Goudsmit, Katarina Radošević

**Affiliations:** 1 Crucell Vaccine Institute, Janssen Center of Excellence for Immunoprophylaxis, Crucell Holland BV, Leiden, the Netherlands; 2 Clinical Immunology, Crucell Holland BV, Leiden, the Netherlands; Public Health Agency of Canada, Canada

## Abstract

Current influenza vaccines are believed to confer protection against a narrow range of virus strains. The identification of broadly influenza neutralizing antibodies (bnAbs) has triggered efforts to develop vaccines providing ‘universal’ protection against influenza. Several bnAbs were isolated from humans recently vaccinated with conventional influenza vaccines, suggesting that such vaccines could, in principle, be broadly protective. Assessing the breadth-of-protection conferred to humans by influenza vaccines is hampered by the lack of *in vitro* correlates for broad protection. We designed and employed a novel human-to-mouse serum transfer and challenge model to analyze protective responses in serum samples from clinical trial subjects. One dose of seasonal vaccine induces humoral protection not only against vaccine-homologous H1N1 challenge, but also against H5N1 challenge. This heterosubtypic protection is neither detected, nor accurately predicted by *in vitro* immunogenicity assays. Moreover, heterosubtypic protection is transient and not boosted by repeated inoculations. Strategies to increase the breadth and duration of the protective response against influenza are required to obtain ‘universal’ protection against influenza by vaccination. In the absence of known correlates of protection for broadly protective vaccines, the human-to-mouse serum transfer and challenge model described here may aid the development of such vaccines.

## Introduction

Influenza virus infections are a major public health concern, with seasonal epidemics and occasional pandemics causing significant morbidity and mortality [1]. The main preventive countermeasure is vaccination. Current influenza vaccines primarily induce antibodies that bind to the globular head domain of the major viral surface glycoprotein, hemagglutinin (HA) and interrupt the viral life cycle by blocking attachment to sialic acid receptors on host cells [2]–[6]. However, as most of the globular head domain is highly variable, antibodies targeting this region are typically highly strain-specific. In the past five years, human antibodies have been isolated that target the stem region of HA instead of the globular head and that neutralize influenza viruses by blocking viral entry rather than attachment to the host cell [7]–[12]. As their epitopes are highly conserved among various influenza virus subtypes, these stem-binding antibodies have heterosubtypic neutralizing activity. Studies conducted after the initial identification of these broadly neutralizing antibodies (bnAbs) have shown that they are less rare than initially believed [13] and that infection with the 2009 pandemic H1N1 virus frequently elicited B cells producing stem-reactive antibodies [14]–[16]. Importantly, various reports indicate that B cells producing bnAbs were also induced in humans following vaccination with the 2009 pandemic H1N1 influenza vaccine [15], [17]–[20], which supports the possibility of designing vaccines that elicit bnAbs and provide broad protection against influenza. Various approaches to the design of such ‘universal’ vaccines are being explored, such as the creation of ‘headless’ HA immunogens [21], [22] and novel immunization strategies [23], [24]. The fact that several bnAbs were isolated from humans recently vaccinated with seasonal vaccines [7], [8], [10]–[12], [25] suggests that conventional trivalent influenza vaccines are also able to induce bnAbs and could, in principle, confer heterosubtypic protection. However, assessing the breadth of protection provided by such vaccines, or any (novel) vaccine modality, in humans is complicated by the fact that the only established *in vitro* correlate of protection exclusively considers the level of antibodies that prevent attachment. We have designed a novel human-to-mouse serum transfer and challenge model to overcome this limitation. Transfer of post-vaccination serum protects mice against vaccine homologous H1N1 challenge, which is strongly correlated to hemagglutination inhibiting antibody titers. We further show that the seasonal vaccine induces protection not only against vaccine-homologous H1N1 challenge, but also, albeit transiently, against heterosubtypic H5N1 challenge.

## Results

### Design of human-to-mouse serum transfer and challenge model

Human serum samples, collected before and after vaccination, were obtained from a vaccine safety trial in which 25 healthy volunteers received repeated injections with the standard dose (15 µg HA of each strain per vaccination) of seasonal trivalent influenza vaccine that included influenza strains A/California/07/2009 (H1N1), A/Victoria/210/2009 (H3N2), and B/Brisbane/60/2008. The vaccine induced a humoral immune response that fulfilled the regulatory criteria for seasonal vaccine immunogenicity (Fig. S1). To assess the humoral protective activity elicited by the vaccine, the serum samples were analyzed for their protective ability in lethal H1N1 and H5N1 mouse challenge models as well as for the presence of H1N1 and H5N1 specific antibodies using various immunological *in vitro* assays.

**Figure 1:**
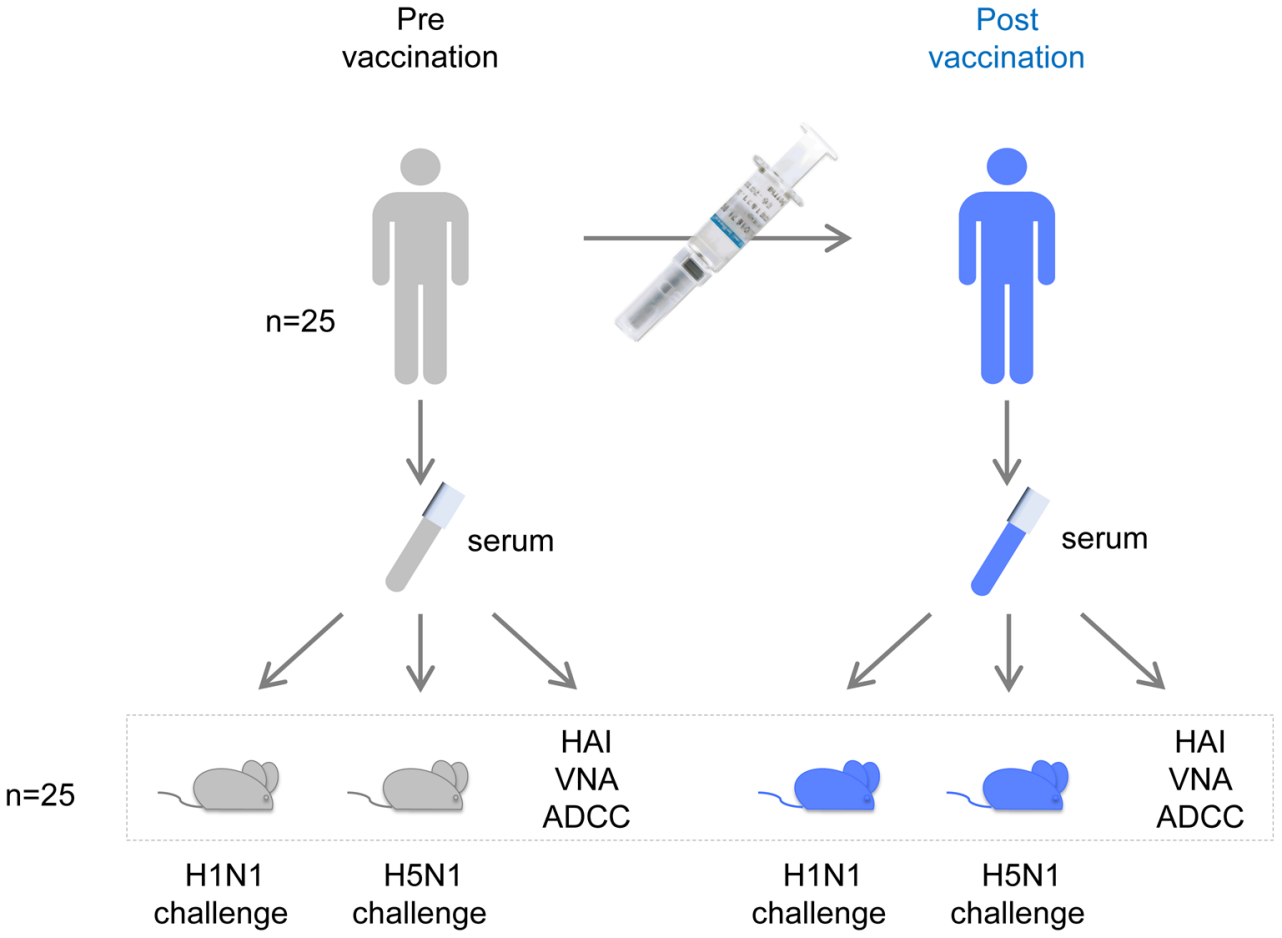
Schematic representation of study design. In a safety and immunogenicity clinical trial (Inf-V-A017), serum was obtained from 25 human subjects before and 4 weeks after vaccination (pre- and post- vaccination serum). To assess the protective activity of the sera, 400 µl of each serum sample was transferred to each of two individual mice which were challenged 24 h later with 25×LD_50_ of vaccine homologous A/Netherlands/602/2009 (H1N1) or heterosubtypic A/Hong Kong/156/97 (H5N1) virus, respectively. In parallel, human sera were characterized for virus-specific immune responses using the hemagglutination inhibition (HAI) assay, virus neutralization assay (VNA), and antibody dependent cellular cytotoxicity (ADCC) assay.

For the challenge studies, we employed a novel serum transfer model in which each individual human serum sample is transferred to an individual mouse before challenge with influenza virus (Fig. 1). In this way, a substantial number of serum samples can be evaluated in a single challenge study while retaining sufficient statistical power. In addition, transferring individual serum samples (as opposed to pooled serum) enables the monitoring of changes in immune response and protective ability of the serum from individual donors over time.

### Seasonal vaccine induces heterosubtypic humoral protection

**Figure 2:**
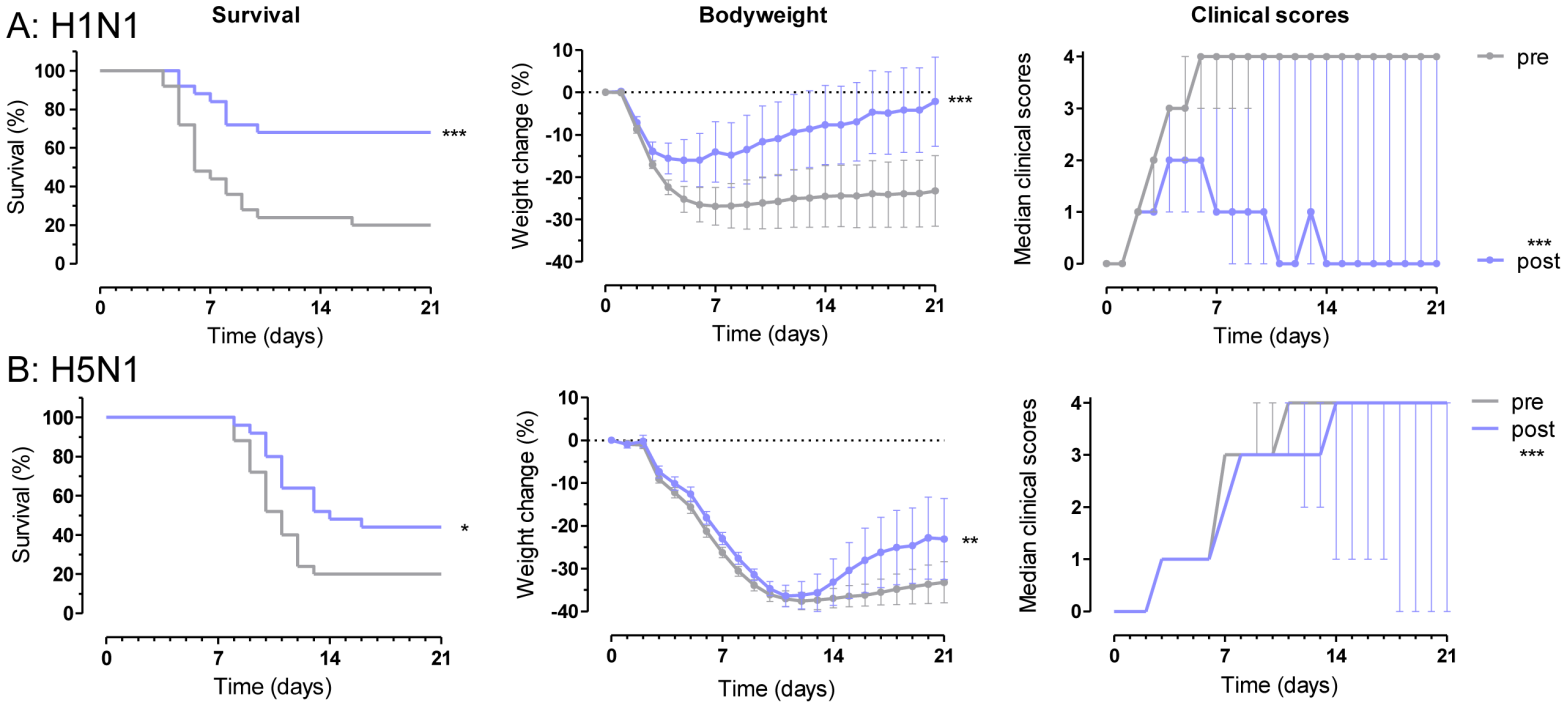
Serum from humans immunized with seasonal influenza vaccine protects mice against vaccine homologous H1 and heterosubtypic H5 influenza challenge. Kaplan-Meier survival curves (left), mean bodyweight change (middle) and median clinical score (right) graphs are shown for mice that received either pre- or post-vaccination serum (grey and blue lines, respectively) following lethal challenge with (**A**) H1N1 or (**B**) H5N1 virus. Error bars indicate 95% confidence interval (bodyweight) or interquartile range (clinical scores). Average bodyweight loss and median clinical score data are presented with last observation carried forward for mice that succumb to infection. Post-vaccination serum confers significant protection against H1N1 and H5N1 influenza challenge relative to pre-vaccination serum. p<0.05 = *, p<0.01 = **, p<0.001 = ***.

In the current study, we transferred 400 µl serum drawn from each volunteer immediately prior to and one month after vaccination to individual mice and subsequently challenged these mice with mouse-adapted A/Netherlands/602/2009 (H1N1 virus; closely related to the H1N1 component of the vaccine) or A/Hong Kong/156/97 (H5N1) virus. As shown in Fig. 2, a significantly larger proportion of mice that received post-vaccination serum survived challenge with vaccine-homologous A/Netherlands/602/2009 (H1N1) virus compared to mice that received pre-vaccination serum (68% vs 20%, sign test p<0.05), demonstrating that the vaccine induced a humoral response that was protective against a closely related virus. The post-vaccination serum also conferred significantly higher protection against heterosubtypic H5N1 challenge than pre-vaccination serum (signed-rank test p = 0.031), albeit just significant. This finding demonstrates the ability of seasonal influenza vaccine to elicit a humoral immune response in humans that mediates cross-reactive, heterosubtypic protection.

Bodyweights and clinical scores show that serum transfer does not completely prevent disease in the recipient mice, but the significant reductions in bodyweight loss and clinical disease symptoms confirm the protective effect of post-vaccination human sera in both challenge models (Fig. 2). The survival of a few mice that received pre-vaccination serum may reflect previous exposure of the corresponding serum donors to related influenza strains, or (as this explanation is unlikely for H5N1) the presence of cross-reactive antibodies in serum elicited by previous exposures to divergent influenza viruses. Another possible explanation might be a low level of non-specific protection by human serum in the model.

### In vitro immunogenicity assays are correlated with homologous protection but fail to predict heterosubtypic protection

**Figure 3:**
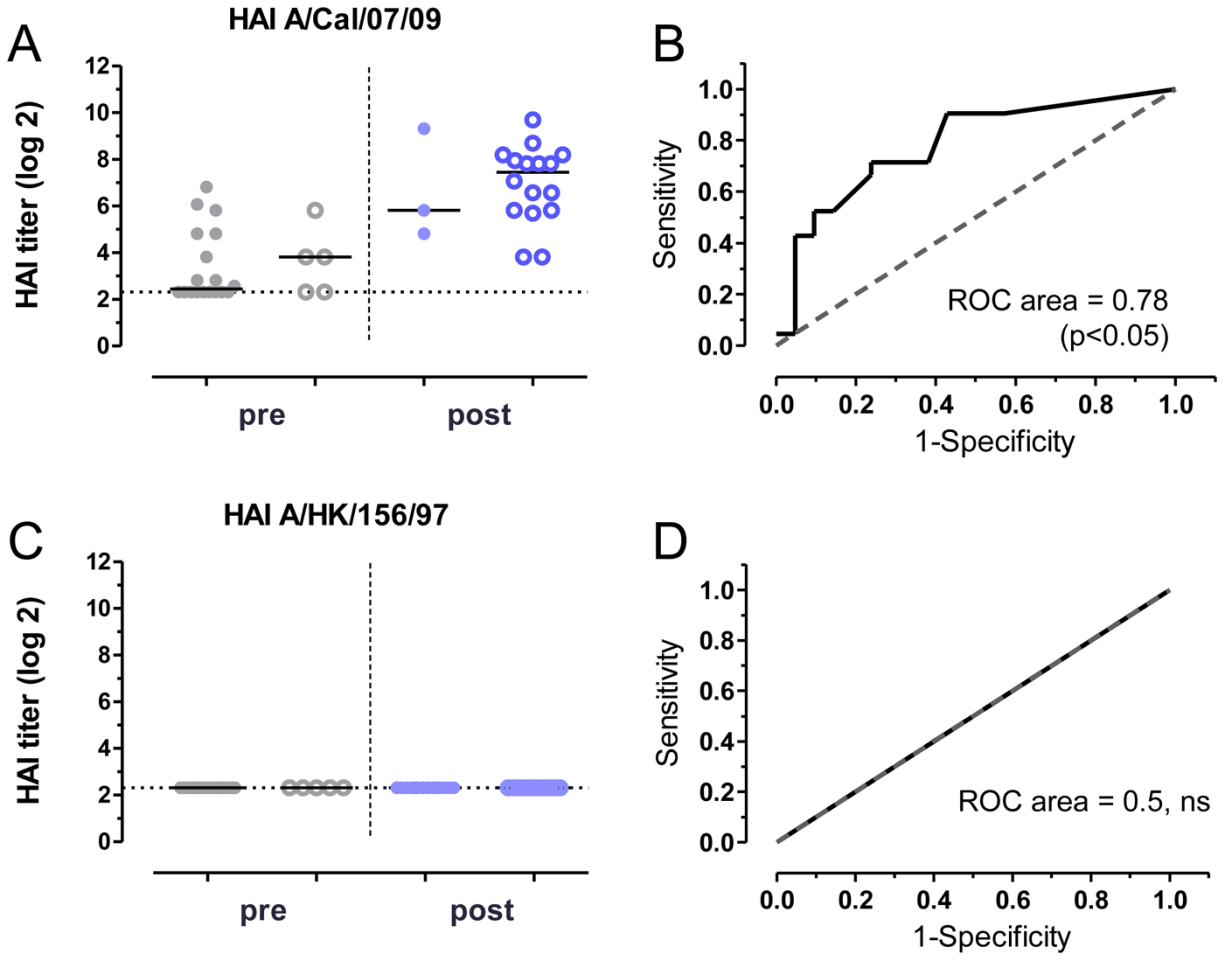
Serum HAI titers correlate with protection against H1N1 but not H5N1 challenge. HAI titers against (**A**) A/California/07/2009 (H1N1) and (**C**) A/Hong Kong/156/97 (H5N1) were determined. Titers for mice that survive challenge (open circles) and mice that succumb to infection (closed circles) are presented separately for pre-vaccination (pre) and post-vaccination (post) sera. Assay background level is indicated with dashed line in panels A and C. Vaccine homologous H1 HAI titers are higher in post-vaccination serum (p<0.001, t-test), while H5 HAI titers are undetectable. Receiver-operator characteristic (ROC) curves were calculated to assess the correlation (**B**) between HAI titers against A/California/07/2009 (H1N1) virus and survival following A/Netherlands/602/2009 (H1N1) challenge, and (**D**) between HAI titers against A/Hong Kong/156/97 (H5N1) virus and survival following A/Hong Kong/156/97 (H5N1) challenge. H1N1 HAI correlates with protection against homologous H1N1 challenge (ROC area = 0.78, p<0.05, 95% confidence interval (CI) 0.64–0.92). ns, not significant.

The immunogenicity and protective ability of influenza vaccines are generally estimated from the results of the *in vitro* hemagglutination inhibition (HAI) assay. Therefore, we determined the HAI titers of the human serum samples against homologous A/California/07/2009 (H1N1) and heterosubtypic A/Hong Kong/156/97 (H5N1) viruses, and performed correlation analysis of the HAI titers with survival of the mice following the corresponding challenge, using the Receiver-Operator Characteristic (ROC) curve method (Fig. 3). The HAI titers against A/California/07/2009 (H1N1) virus increased with vaccination (p<0.001) and correlated with survival after A/Netherlands/602/2009 (H1N1) challenge (ROC area 0.78, p<0.05), confirming that HAI is an *in vitro* correlate of homologous protection in our passive transfer model. No HAI titers against A/Hong Kong/156/97 (H5N1) virus could be detected in any of the serum samples. This is according to expectations, given that the HAI assay only detects antibodies directed to the highly variable globular head of the HA molecule, so that neither vaccination with vaccine containing A/California/07/2009 (H1N1) nor previous exposure to circulating viruses is likely to induce H5-specific antibodies. This result does however illustrate the inadequacy of the HAI assay to estimate the protective ability of a vaccine against divergent influenza strains.

**Figure 4:**
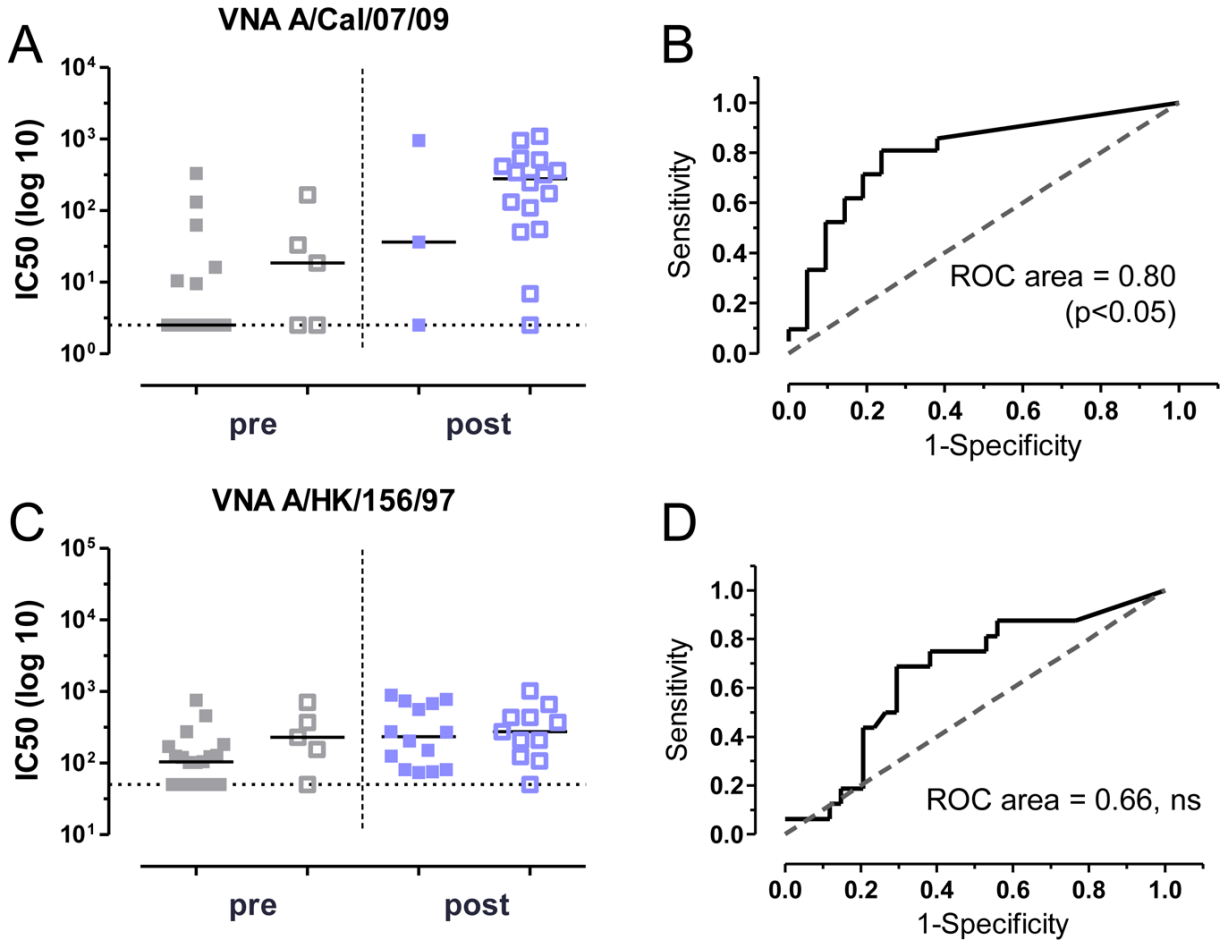
Serum VNA titers correlate with protection against H1N1 but not H5N1 challenge. VNA titers against (**A**) A/California/07/2009 (H1N1) and (**C**) A/Hong Kong/156/97 (H5N1) were determined. Titers for mice that survive challenge (open squares) and mice that succumb to infection (closed squares) are presented separately for pre-vaccination (pre) and post-vaccination (post) sera. Assay background level is indicated with dashed line in panels A and C. Vaccine homologous H1 VNA titers are higher in post-vaccination serum (p<0.001, t-test). H5 VNA titers are detectable in some pre-vaccination samples and are higher in post-vaccination serum (p<0.001, t-test). ROC curves were calculated to assess the correlation (**B**) between H1N1 A/California/07/2009 VNA and protection against H1N1 A/Netherlands/602/2009 challenge and (**D**) between H5N1 A/Hong Kong/156/97 VNA and protection against H5N1 A/Hong Kong/156/97 challenge. H1N1 VNA correlates with protection against homologous H1N1 challenge (ROC area = 0.80, p<0.05, 95% CI 0.66–0.94). The positive correlation between H5 VNA and H5 protection does not reach statistical significance (ROC area = 0.66, 95% CI = 0.496–0.82).

Therefore, we also determined the neutralizing activity of the serum samples against A/California/07/2009 (H1N1) and A/Hong Kong/156/97 (H5N1) viruses using a virus neutralization assay (VNA) (Fig. 4). In contrast to the HAI assay, the VNA assay is not selectively sensitive for the activity of antibodies that target the head region of HA, but is also able to detect the activity of any neutralizing antibody directed against HA, or even neuraminidase, the other major surface glycoprotein of influenza viruses. Like the HAI titers, the VNA titers against A/California/07/2009 (H1N1) increased following vaccination (p<0.001) and correlated significantly with protection (ROC area 0.80, p<0.05). This is not surprising, considering that the antibodies targeting the head domain of HA (and thus capable of mediating HAI) are a major part of the total virus-neutralizing antibody response [26], [27]. Interestingly, neutralizing antibodies against more conserved epitopes were also present, as indicated by the fact that neutralizing titers against A/Hong Kong/156/97 (H5N1) virus were detectable in most serum samples (Fig. 4C). Although these titers slightly increased after vaccination (p<0.001), the correlation between these VNA titers and survival following H5N1 challenge was not statistically significant.

**Figure 5:**
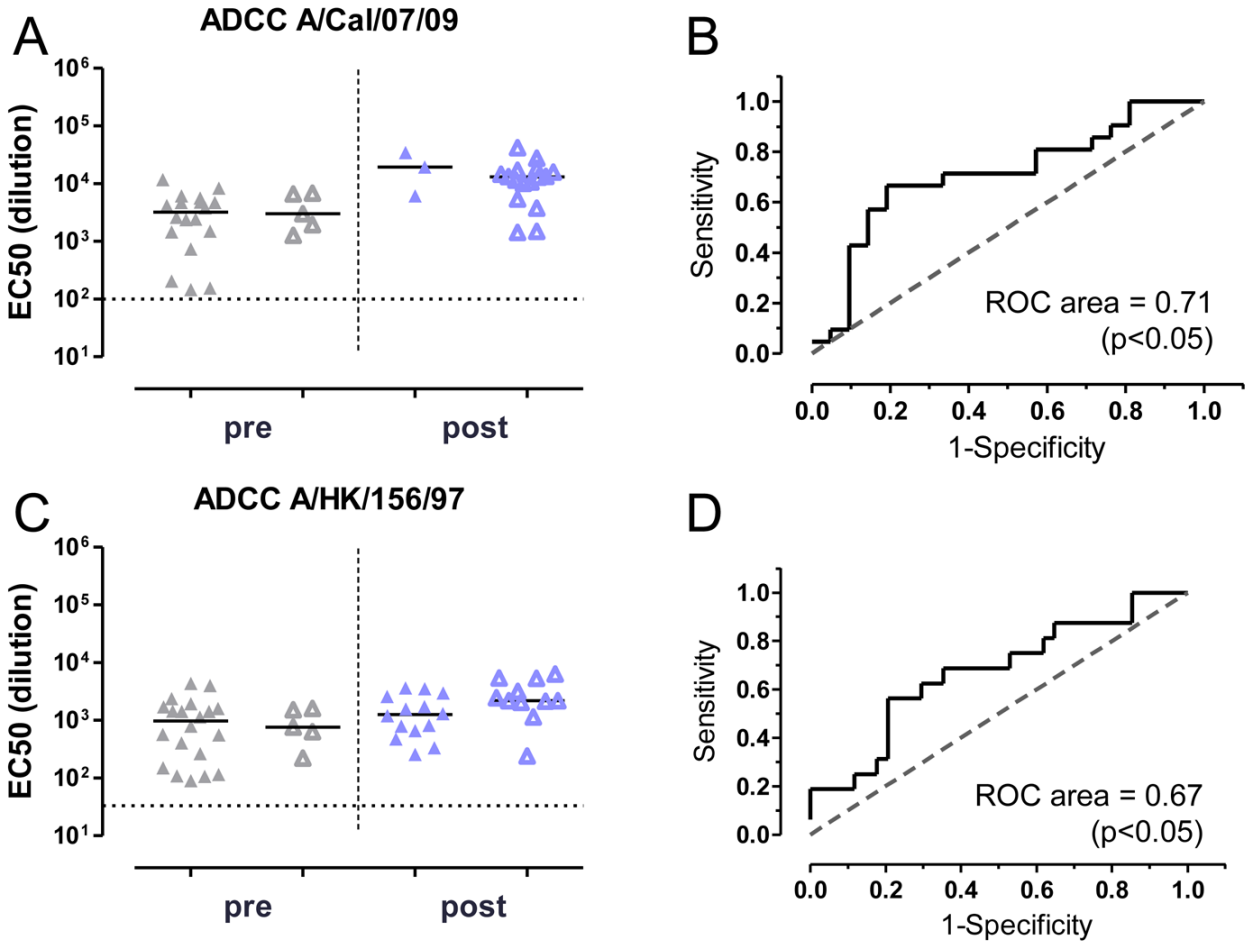
Serum ADCC titers weakly correlate with protection against H1N1 and H5N1 challenges. ADCC titers against (**A**) A/California/07/2009 (H1N1) and (**C**) A/Hong Kong/156/97 (H5N1) were determined. Titers for mice that survive challenge (open triangles) and mice that succumb to infection (closed triangles) are presented separately for pre-vaccination (pre) and post-vaccination (post) sera. Assay background level is indicated with dashed line in panels A and C. Vaccine homologous H1 ADCC titers are higher in post-vaccination serum (p<0.001, t-test). H5 ADCC titers are detectable in pre-vaccination samples and increase significantly after vaccination (p<0.001, t-test). ROC curves were calculated to assess the correlation (**B**) between H1N1 ADCC titer and protection against homologous H1N1 challenge (ROC area = 0.71, p<0.05, 95% CI 0.55–0.87) and (**D**) between H5N1 ADCC titer and protection against H5N1 challenge (ROC area = 0.67, p<0.05, 95% CI 0.505–0.84).

Recently, cross-reactive antibody-dependent cellular cytotoxicity (ADCC) has been implicated as a mechanism that reduces the severity of divergent influenza virus infections [28]. Furthermore, ADCC has been shown to contribute to the *in vivo* antiviral activity of broadly neutralizing HA stem-binding antibodies [11], [12]. We therefore assessed the levels of virus-specific ADCC in human serum before and after vaccination, and analyzed the correlation with survival in the mouse challenge model (Fig. 5). Detectable ADCC titers against A/California/07/2009 (H1N1) and A/Hong Kong/156/97 (H5N1) were present in pre-vaccination serum samples, and both increased after vaccination (p<0.001) (Fig. 5A, C). Virus-specific ADCC titers correlated with survival after challenge with A/Netherlands/602/2009 (H1N1) (ROC area 0.71, p<0.05) and A/Hong Kong/156/97 (H5N1) (ROC area 0.67, p<0.05) (Fig. 5B, D). While titers increased following vaccination, there was a large overlap in titers between protected animals and those that succumbed to infection, which ruled out accurate prediction of the challenge outcome based on ADCC level. Like HAI and VNA titers, ADCC titer is therefore not suitable as a correlate of protection assay.

### Heterosubtypic protection is not boosted by subsequent immunizations

**Figure 6:**
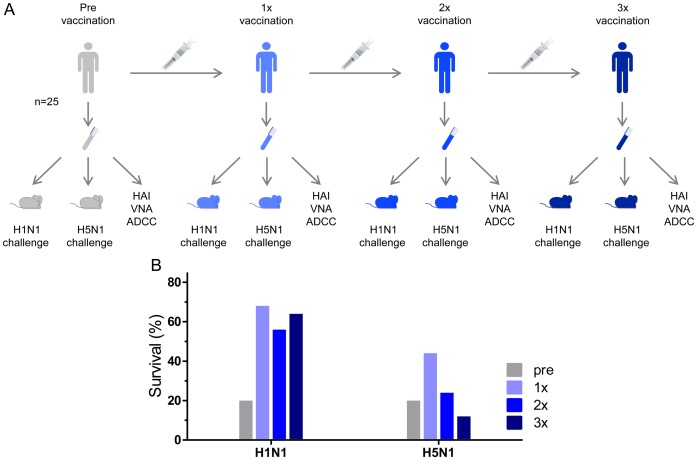
Heterosubtypic protection by post-vaccination serum is not boosted by subsequent immunizations and is lost after the second vaccination. (**A**) Serum was obtained from 25 human subjects immediately prior to the first vaccination and 4 weeks after each of three vaccinations (pre-vaccination, 1×, 2×, and 3×, respectively). (**B**) Human-to-mouse serum transfer was used to assess humoral protection against vaccine homologous H1N1 A/Netherlands/602/2009 and heterosubtypic H5N1 A/Hong Kong/156/97 influenza viruses. To assess the protective activity of the sera, 400 µl of each serum sample was transferred to each of two individual mice which were challenged 24 h later with 25×LD_50_ of vaccine homologous A/Netherlands/602/2009 (H1N1) or heterosubtypic A/Hong Kong/156/97 (H5N1), respectively. All post-vaccination groups show comparable protection against H1N1 challenge. There is a significant difference in protection against H5N1 among different vaccination groups (exact logistic regression, p = 0.035).

Our novel serum transfer model provides a tool for studying heterosubtypic protection by seasonal influenza vaccines despite the lack of a predictive *in vitro* correlate for the protection of serum-recipient mice following heterosubtypic (H5N1) challenge. In order to determine whether the level of heterosubtypic protection can be enhanced by repeated vaccinations with the same vaccine, we applied the human-to-mouse serum transfer and challenge method to serum samples drawn one month after a second vaccination and one month after a third vaccination (Fig. 6A). Whereas the level of protection against H1N1 challenge conferred by serum samples drawn after the second and third vaccinations was comparable to that of the serum samples drawn after the first vaccination, the protective effect against H5N1 challenge of serum samples drawn after the first vaccination was lost one month after the second vaccination (Fig. 6B, Fig. S2B, S2C). This finding was corroborated with the change in mouse bodyweight after challenge when analyzed for serum samples from individual donors (Fig. S2D).

Neither the H1N1-specific nor the H5N1-specific HAI, VNA and ADCC titers do not increase significantly after the second and third vaccinations when compared to the first vaccination (Fig. S3). The homologous H1N1 *in vitro* assay results are in agreement with H1N1 *in vivo* challenge data and both indicate that repeated vaccination with the seasonal vaccine at 1-month intervals does not significantly enhance the homologous humoral protection obtained after single vaccination. In contrast, the heterosubtypic H5N1 protection and its transient nature detected *in vivo* using the serum transfer and challenge model are not predicted by the *in vitro* assays (Fig. 6B, Fig. S3).

## Discussion

Using a novel human serum transfer and mouse influenza challenge model, we demonstrate that passive protection of mice with serum from humans vaccinated with trivalent seasonal influenza vaccine recapitulates relevant features of human protection against homologous influenza. Moreover, vaccination of healthy adults not only induces humoral protection against a vaccine-homologous H1N1 virus, but also against a highly pathogenic avian strain of the H5N1 subtype. This finding is remarkable because current seasonal influenza vaccines are generally believed to induce protective immunity only to virus strains closely related to the ones included in the vaccine.

This belief is in part based on use of the HAI assay to assess the immunogenicity and protective ability of influenza vaccines. Although this assay is a good indicator of the virus-neutralizing activity of antibodies targeting the head region of the HA glycoprotein, and a reasonable correlate of protection against vaccine-homologous influenza strains [29], it does not detect neutralizing antibodies targeting the stem of the HA molecule or other antibody-mediated protective mechanisms such as ADCC. Therefore, it underestimates the protective ability of an influenza vaccine, in particular against phylogenetically distant strains. In line with this notion, a recent systematic meta-analysis has shown that seasonal vaccines can in fact provide cross-protection against non-matching circulating strains [30].

Interestingly, the heterosubtypic H5N1 protection observed in our study was not boostable by repeated vaccination, but waned and was lost within two months. As all volunteers received multiple doses of vaccine, we do not know whether the heterosubtypic protective activity of the sera following vaccination is intrinsically short-lived, or was abrogated by the subsequent vaccination. A similar transient cross-reactive antibody response was observed by Corti *et al.* [11] in a donor that was first infected with A/California/04/2009 (H1N1) and vaccinated two months later with a vaccine containing the same virus. Whereas most HA-specific antibodies derived from B cells isolated one week after infection bound to both H1 and H5 (89.7%), antibodies derived from B cells 1 week after subsequent vaccination were largely vaccine H1-specific (98.2%). Analogous to the model by Li *et al.* [18], in which vaccination or infection with a novel (pandemic) virus strain is proposed to induce more cross-reactive memory B cells specific for conserved epitopes than vaccination or infection with a seasonal virus, one could imagine that the first shot of a seasonal vaccine re-activates more cross-reactive memory B cells than subsequent immunizations with the same vaccine and that these repeated vaccinations shift the response towards strain-specific epitopes. Thus, antibodies to the conserved region of influenza HA could potentially be involved in the observed heterosubtypic protection.

To assess the immune responses of the trial participants *in vitro*, we used the HAI, VNA and ADCC assays. These commonly used assays were selected because each measures a different mechanism of viral neutralization and their combination presumably enables quantification of broad and effective humoral immune response. Serum titers of antibodies measured with all three *in vitro* assays correlated with protection, as characterized by survival and reductions in bodyweight loss and clinical scores, following homologous H1N1 challenge. Thus, we confirm that HAI, which is a known *in vitro* correlate of protection against homologous influenza in humans, is also identified as a correlate of homologous protection in passive transfer. Only ADCC titers correlated significantly with protection against H5N1 (albeit too weakly to predict challenge outcome). Whereas the serum-mediated H5N1 protection and its transient nature were revealed using the serum transfer and challenge model, these effects would have been missed based on results of the *in vitro* assays only. These findings underscore that different antibodies with different mechanisms of action play a role in protection against different influenza strains. As a consequence, no single *in vitro* assay may accurately assess the protective activity, particularly of novel vaccine modalities designed to confer broad protection. Our novel serum transfer and challenge model can be used to further dissect the humoral components that contribute to protection.

Mouse passive transfer models have been used previously to assess the protective activity of human serum samples [15], [31]–[33]. However, the design applied here in which each individual human serum sample is transferred to an individual mouse allows for the evaluation of a substantial number of serum samples in a single challenge study while retaining sufficient statistical power. Furthermore, this design enables not only the evaluation of protective responses per group and per time point but also monitoring of changes in the immune response and protective ability of serum from individual donors at different time points. In this study, the humane endpoint following challenge was – similar to the mouse LD_50_ determinations for both challenge viruses – defined on a clinical score as a measure of discomfort, rather than on a certain percentage of bodyweight loss. However, other humane endpoints will be equally suited for the read out in serum transfer challenge experiments as long as they match with the endpoint used in the LD_50_ determination of the challenge virus.

Clearly, protection of mice after passive serum transfer and subsequent challenge will not translate one-to-one to protection in humans, as factors such as the amount of serum transferred and the dose of challenge virus used will influence the absolute level of protection in mice. Moreover, the overall protective ability of an influenza vaccine may be underestimated by measuring only the protective ability of its humoral component. However, this approach could, for example, be used to evaluate the protective abilities of novel (universal) vaccines in phase I trials and enable the selection of the most promising candidates for further clinical studies, in the absence of known *in vitro* correlates of protection.

In conclusion, using a human serum transfer and mouse challenge model we demonstrate that single immunization with a seasonal influenza vaccine is able to elicit an H5N1 protective humoral response in humans. This protective response is not boosted by subsequent immunization with the same vaccine and wanes within months. We conclude that strategies to increase the breadth and duration of the protective response elicited by conventional vaccines, such as adjuvants [34]–[36] or novel vaccination schedules involving DNA priming [37], [38], or altogether novel vaccine modalities are required to obtain ‘universal’ protection against influenza through vaccination. The human-to-mouse serum transfer and challenge model described here can assist the development of either of these strategies.

## Materials and Methods

### Human sera

Serum was obtained from a vaccine safety and immunogenicity trial (INF-V-A017, EudraCT #2012-001693-28). Twenty five healthy male and female adult human subjects (18–50 years of age on the day of enrolment) were randomly selected to receive a vaccination regimen consisting of three consecutive intramuscular vaccinations with Inflexal V, a trivalent seasonal influenza vaccine of the 2011/2012 composition (H1N1 A/California/07/2009, H3N2 A/Victoria/210/2009, and B/Brisbane/60/2008; 15 µg HA per strain per vaccination). Repeated vaccination was performed to evaluate boosting ability of the seasonal vaccine. The study was performed between influenza seasons to minimize the risk of influenza infection during the study, which could affect read-out. Subjects who had previously been vaccinated with a seasonal influenza vaccine for the 2011–2012 were excluded from participation. The study was approved by the ‘Universitair Ziekenhuis Antwerpen Comité voor Medische Ethiek’ ethical review board. Written informed consent was obtained from all subjects prior to enrolment.

### Influenza challenge

Human serum (400 µl) was transferred to recipient mice (female BALB/c, 6–8 weeks old) via intraperitoneal injection 24 hours prior to challenge with influenza virus. On the day of challenge, a pre-challenge mouse blood sample was obtained via submandibular bleeding to assess for successful human serum transfer *(figure S2A)*. Successful transfer was assessed on the basis of the correlation between rH1 A/Californai/07/2009 specific antibody titers in the human serum and mouse pre-challenge serum. Recipient titers are tightly correlated with the corresponding human titer, and approximately 8-fold lower due to serum dilution. When titers in serum recipient mice were >100 fold below the corresponding human serum titers this was considered as a failed transfer (dashed line). Mice for which the serum transfer was unsuccessful were excluded from the correlate of protection analysis. Stocks of H1N1 A/Netherlands/602/2009 (Viroclinics) and H5N1 A/Hong Kong/156/97 (Central Veterinary Institute, Wageningen University) were grown on embryonated chicken eggs. Anaesthetized mice were challenged intranasally with 25×LD_50_ virus in a total volume of 50 µl. Groups of mice (n = 8) receiving either PBS or a broadly protective monoclonal antibody (CR6261, 15 mg/kg in PBS) intramuscularly (H1N1 study) or intravenously (H5N1 study) were used as negative and positive controls for challenge models, respectively (see figure S2B and S2C). Challenges were considered valid when there was a statistically significant difference in survival proportion (Fisher's exact-test, 2-sided) between the control groups *(data not shown)*. Bodyweight and clinical score were monitored daily for up to 21 days or until a humane endpoint (clinical score 4), to limit animal discomfort. Clinical scores were defined as: 0 = no clinical signs, 1 = rough coat, 2 = rough coat, less reactive, passive during handling, 3 = rough coat, rolled up, labored breathing, passive during handling, 4 = rough coat, rolled up, labored breathing, unresponsive. All mouse studies were approved by DEC Consult (Independent ethical institutional review board).

### Hemagglutination Inhibition Assay

Non-specific agglutination inhibitors were removed from serum samples by overnight incubation with Neuraminidase Cholerae at 37°C (Sigma-Aldrich; St. Louis, Missouri, USA, 50mUnits, 100 µl/50 µl serum), which was subsequently inactivated by incubation with sodium citrate (75 µl, 2.5%) for 30 minutes at 56°C. PBS (25 µl) (Gibco, Life Technologies; Carlsbad, California, USA, pH 7.4) and human red blood cells (from Human Whole Blood Type O, 250 µl, 2.5% in PBS; Seralab; West Sussex, UK) were added to obtain a final sample dilution of 1∶10. Samples were incubated for 1 hour at room temperature and spun down at 10.000× g for 5 minutes. Twofold serial dilutions of the supernatant in PBS were prepared in duplicate, mixed by agitation with 8 HA units (25 µl) of H1N1 A/California/07/2009 (reassortant NYMC X-181) or H5N1 A/Hong Kong/156/97 (reassortant rgPR8-H5N1) virus and incubated for 1 hour at room temperature. Confirmed positive and negative sera were used as assay controls. Human red blood cells (50 µl, 0.75% in PBS) were added to all wells, mixed by agitation and incubated for 1 hour at room temperature. The HAI titer of a given serum sample was determined as the reciprocal of the highest dilution where no hemagglutination inhibition was observed.

### Virus Neutralization Assay

Madin-Darby Canine Kidney (MDCK) cells were seeded in a 96-well plate at 15.000 cells/well in growth medium (DMEM containing 200 mM L-glutamine, 3 µg/ml trypsin and 1% (w/v) penicillin/streptomycin stock solution, all GIBCO/Invitrogen) and allowed to attach for at least 3 hours. Duplicate serial dilutions of heat-inactivated (30 minutes at 56°C) serum samples (0.01–20%) were prepared in DMEM with or without trypsin/EDTA (0.6% of a 0.05% stock solution) and mixed with 112 TCID_50_ of H1N1 A/California/07/2009 (reassortant NYMC X-181) or 200 TCID_50_ of the H5N1 A/Hong Kong/156/97 (reassortant rgPR8-H5N1) virus per sample, respectively, for 1 hour at 37°C, 10% CO_2_. Mixes were subsequently added to the MDCK cells and incubated for 18 hours at 37°C, 10% CO_2_. Cells were fixed with 80% acetone, labeled with mouse anti-NP (H16-L10-4R5, produced in house), followed by goat anti-mouse HRP-coupled antibody (KPL, Gaithersberg, USA) for one hour each. TMB substrate (H1N1) or ABTS substrate (H5N1) (Roche, Basel, Switzerland) was added, and absorbance was read in a BioTek reader (PerkinElmer) after 5–15 minutes. Monoclonal antibody CR9114 (human IgG1, produced in house) and macaque serum were used as a positive and negative controls, respectively. Samples without detectable neutralization at the lowest dilution are indicated as the lowest dilution (i.e. background level). The IC_50_ values were calculated after 4-parameter logistic curve fit.

### Antibody Dependent Cellular Cytotoxicity assay

A549 cells were seeded in clear and white bottom 96-well plates (15.000 cells/well) and were infected the next day with 3.6×10^3^ TCID_50_/well of H1N1 A/California/07/2009 (reassortant NYMC X-181) or H5N1 A/Hong Kong/156/97 (reassortant rgPR8-H5N1) virus in infection medium (DMEM, containing 200 mM L-glutamine, 0.1 µg/ml trypsin, and 1% (w/v) penicillin/streptomycin stock solution, all GIBCO/Invitrogen). Plates were incubated at 37°C, 10% CO_2_ for 18–20 hours. The clear bottom plate was subjected to direct immunofluorescence staining to confirm infection levels. The cells were fixed with 80% acetone, followed by labeling with AlexaFluor488-labeled anti-NP staining (hybridoma H16-L10-4R5) and DAPI to stain cell nuclei (*results not shown*). White bottom plates were used for a commercially available ADCC reporter assay (PROMEGA, G7010). Serum samples were serially diluted in assay buffer (RPMI, 4% ultra-low IgG FBS, 1% (w/v) penicillin/streptomycin stock solution, all GIBCO/Invitrogen) and added to the infected cells after medium was removed. Jurkat reporter cells (9×10^4^/well) stably expressing human FcγRIIIa (V158 variant) and an NFAT response element driving expression of firefly luciferase were added to the infected target cells, and incubated at 37°C for 6 hours. Bio-Glo Luciferase Assay reagent (PROMEGA) was added according to manufacturer's instructions, and luminescence was measured after 10 min using a TriLux plate reader (PerkinElmer). Monoclonal antibody CR9114 (human IgG1, produced in house) and macaque serum were used as positive and negative controls, respectively. Indicated background levels are the lowest dilutions used in the assay. The EC_50_ values were calculated after 4-parameter logistic curve fit.

### rH1 A/California/07/2009 ELISA

Multisorp (Nunc) 96-well flat bottom plates were coated overnight with rH1 A/Californai/07/2009 (Protein Science Inc., 0.5 µg/ml), washed and blocked. Wells were incubated with duplicate serial dilutions of human serum or mouse pre-challenge serum for 1 h at room temperature, and washed. Well were incubated with anti-human IgG (Jackson ImmunoResearch) or anti-mouse IgG (KPL) conjugated to HRP for 1 h at room temperature, washed and developed using OPD substrate (Thermo Fischer Scientific). OD was read at 492 nm using a Powerwave Synergy plate reader (Biotec), and compared to standard curve of CR9114 (produced in house), for calculation of ELISA units using a slope based weighted average approach.

### Statistical Methods

According to the design of the study, serum taken pre- or post-vaccination from each individual donor is transferred to a single mouse. This procedure is done for each challenge strain. The mouse responses corresponding to each human donor are then compared between different time points and summarized across donors (block design). In addition, the survival of mice across donors is correlated with antibody titers measured in human serum using different assays. Since serum from all individual donors was used for the transfer studies, this design allows the conclusions to be generalized to the population of subjects randomized into the clinical trial.

#### Influenza challenge studies

Mouse survival up to day 21 is analyzed with the sign test of the difference in survival state (0 = died, 1 = survived) between each pair of mice receiving the post- and pre-vaccination serum of the same human donor, respectively. Differences in survival duration are calculated similarly but tested with the Wilcoxon signed-rank test. The AUC bodyweight is the area under the curve (AUC) of the change in bodyweight relative to the baseline bodyweight from day 0 up to day 21 after the challenge, with the last observed bodyweight carried forward if a mouse died/was euthanized during the study. On the AUC bodyweights, analysis-of-variance is done with vaccination group and human donor as explanatory factors. Clinical scores are summarized per mouse as percentage of days at each score level and analyzed by cumulative logistic regression with score level, vaccination group and human donor as explanatory factors and mouse as subject factor. In figure 6, survival status is analyzed using exact logistic regression with number of vaccinations and human donor as factors, implicitly matching mouse survival by human serum donor.

#### Correlate of protection analysis

Receiver-Operator Characteristic (ROC) curve methodology is applied for calculating the protection by serum titers. For this purpose, the survival status of each mouse and the corresponding assay titer in human serum samples from the pre- and post-vaccination groups is used to calculate the ROC curve. The area under the ROC curve is calculated and the null hypothesis is tested for equality to 0.50 (no information or no correlation with protection) with an asymptotic Mann-Whitney test.

#### Serological assays

For assays performed on the human serum samples, the log titers at the pre- and post-vaccination visits are compared using analysis-of-variance (ANOVA) with visit and human donor as factors. The post-hoc t-tests are adjusted with a 3-fold Bonferroni correction. Titers at the detection limit are analyzed as censored observations.

## Supporting Information

Figure S1 **The European Medicines Agency (EMA) specifies that seasonal vaccines have to meet at least one of the three following criteria for each of the influenza strains that they contain.** Vaccination should result in seroprotection in at least 70% of healthy adult human subjects (defined as a haemagglutination inhibition (HAI) titer >40), seroconversion in at least 40% of healthy adult human subjects (defined as a >4 fold increase in HAI titer), or an average increase (geometric mean) of the HAI titer >2.5 fold. Plots show the percentage of subjects that reach seroprotection (left) and seroconversion (middle), and the average increase in HAI titer relative to pre-vaccination serum (right). Regulatory thresholds are indicated by a green dashed line. The trivalent virosomal vaccine Inflexal V used in these studies is immunogenic and meets regulatory guidelines for all three influenza strains.
(TIF)

Figure S2 **Influenza challenge after human-to-mouse serum transfer sensitively identifies vaccine induced changes in protective ability at different timepoints and for individual subjects.** (**A**) Reproducible recovery of human antibody titers in pre-challenge serum. Transfer efficiency can be observed by tight correlation between rH1 A/Californai/07/2009 binding antibodies in mouse pre-challenge serum relative to the corresponding human pre- or post-vaccination serum (pre, 1×, 2×, 3×) (grey and blue, respectively) When recipient titers were >100 fold below the corresponding human serum titers this was considered as a failed transfer (dashed line), in which case data were excluded from correlation analysis. (**B, C**) Kaplan-Meier survival curves, mean bodyweight change, and median clinical score are shown from left to right for mice that received pre- or post-vaccination serum (pre, 1×, 2×, 3×) (grey and blue, respectively) following lethal challenge with (**B**) H1N1 or (**C**) H5N1 virus. Error bars indicate 95% confidence interval (bodyweight) or interquartile range (clinical scores). Average bodyweight loss and median clinical score data are presented with last observation carried forward for mice that succumb to infection. (**D**) Extrapolated area under the curve (AUC) bodyweight mouse data are depicted per human subject for pre-vaccination, 1×, 2×, and 3× vaccination serum. The extrapolated AUC bodyweight is the area under the curve (AUC) of the change in bodyweight relative to the baseline bodyweight from day 0 up until day 21 after the challenge. The bodyweight of mice that succumb prior to the end of the study is extrapolated using linear exponential decay based on the first and last recorded bodyweights. Each line represents a single subject. Protection against H1N1 is maintained, while protection against H5N1 wanes and is lost one month after the second vaccination. P<0.05 = *, p<0.01 = **, p<0.001 = ***.
(TIF)

Figure S3 **Virus challenge strain–specific HAI, VNA and ADCC titers remain constant after first immunization.** HAI, VNA and ADCC titers against (**A**) H1N1 A/California/07/2009 and (**B**) H5N1 A/Hong Kong/156/97 are depicted for pre-vaccination serum and sera obtained after 1×, 2×, and 3× vaccinations. Dashed lines indicate background levels in the respective assays. The titers at all three post-vaccination visits are statistically significantly higher (p<0.001) than at the pre-vaccination visit for all assays except for HAI H5N1 where all titers fall below the detection limit.
(TIF)
