# Supplementary figures and images for: Transient Humoral Protection against H5N1 Challenge after Seasonal Influenza Vaccination of Humans
Source: PLoS One. 2014 Jul 30;9(7):e103550. doi: 10.1371/journal.pone.0103550 (PMC4116209; doi:10.1371/journal.pone.0103550)

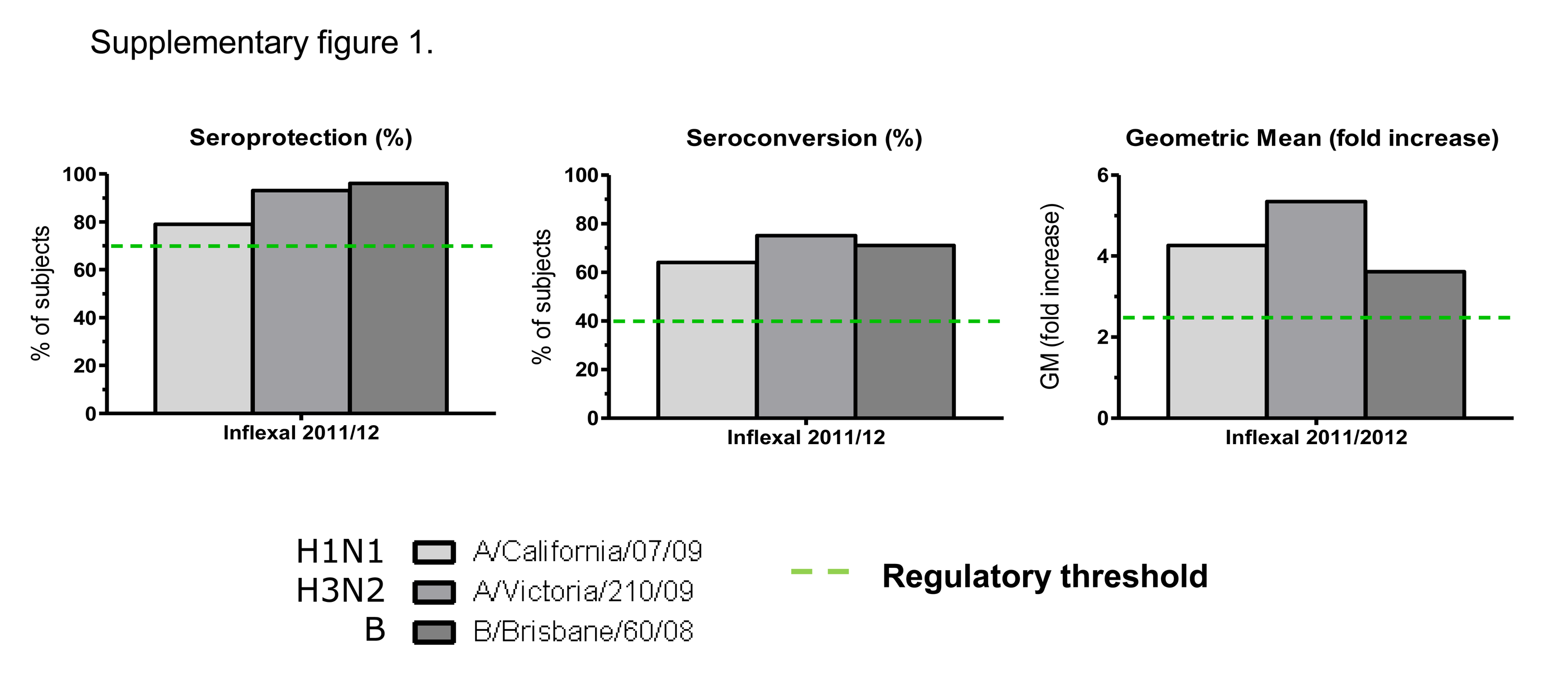

Supplement: Figure S1 — The European Medicines Agency (EMA) specifies that seasonal vaccines have to meet at least one of the three following criteria for each of the influenza strains that they contain. Vaccination should result in seroprotection in at least 70% of healthy adult human subjects (defined as a haemagglutination inhibition (HAI) titer >40), seroconversion in at least 40% of healthy adult human subjects (defined as a >4 fold increase in HAI titer), or an average increase (geometric mean) of the HAI titer >2.5 fold. Plots show the percentage of subjects that reach seroprotection (left) and seroconversion (middle), and the average increase in HAI titer relative to pre-vaccination serum (right). Regulatory thresholds are indicated by a green dashed line. The trivalent virosomal vaccine Inflexal V used in these studies is immunogenic and meets regulatory guidelines for all three influenza strains. (TIF) [file pone.0103550.s001.tif]

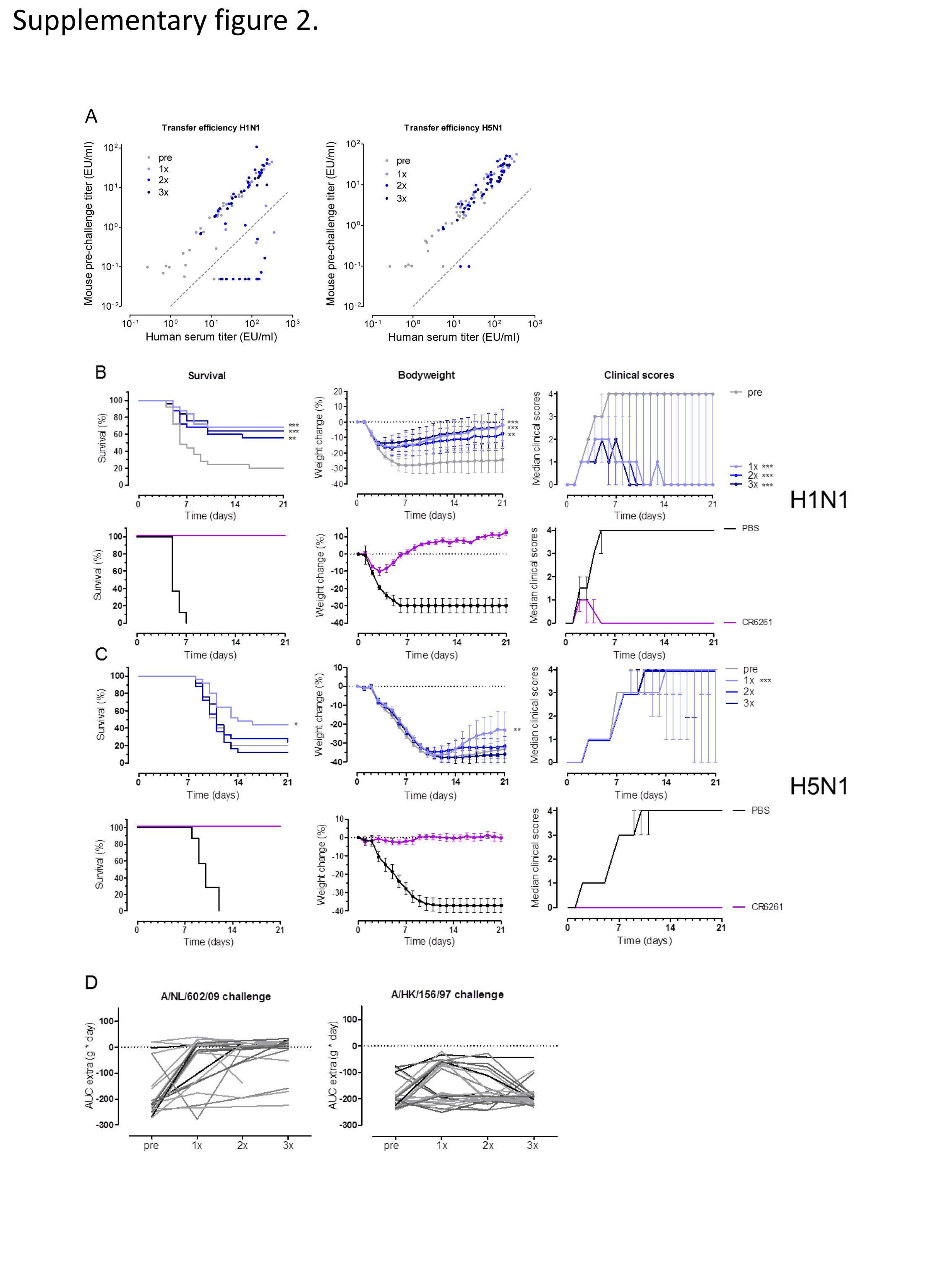

Supplement: Figure S2 — Influenza challenge after human-to-mouse serum transfer sensitively identifies vaccine induced changes in protective ability at different timepoints and for individual subjects. (A) Reproducible recovery of human antibody titers in pre-challenge serum. Transfer efficiency can be observed by tight correlation between rH1 A/Californai/07/2009 binding antibodies in mouse pre-challenge serum relative to the corresponding human pre- or post-vaccination serum (pre, 1×, 2×, 3×) (grey and blue, respectively) When recipient titers were >100 fold below the corresponding human serum titers this was considered as a failed transfer (dashed line), in which case data were excluded from correlation analysis. (B, C) Kaplan-Meier survival curves, mean bodyweight change, and median clinical score are shown from left to right for mice that received pre- or post-vaccination serum (pre, 1×, 2×, 3×) (grey and blue, respectively) following lethal challenge with (B) H1N1 or (C) H5N1 virus. Error bars indicate 95% confidence interval (bodyweight) or interquartile range (clinical scores). Average bodyweight loss and median clinical score data are presented with last observation carried forward for mice that succumb to infection. (D) Extrapolated area under the curve (AUC) bodyweight mouse data are depicted per human subject for pre-vaccination, 1×, 2×, and 3× vaccination serum. The extrapolated AUC bodyweight is the area under the curve (AUC) of the change in bodyweight relative to the baseline bodyweight from day 0 up until day 21 after the challenge. The bodyweight of mice that succumb prior to the end of the study is extrapolated using linear exponential decay based on the first and last recorded bodyweights. Each line represents a single subject. Protection against H1N1 is maintained, while protection against H5N1 wanes and is lost one month after the second vaccination. P<0.05 = *, p<0.01 = **, p<0.001 = ***. (TIF) [file pone.0103550.s002.tif]

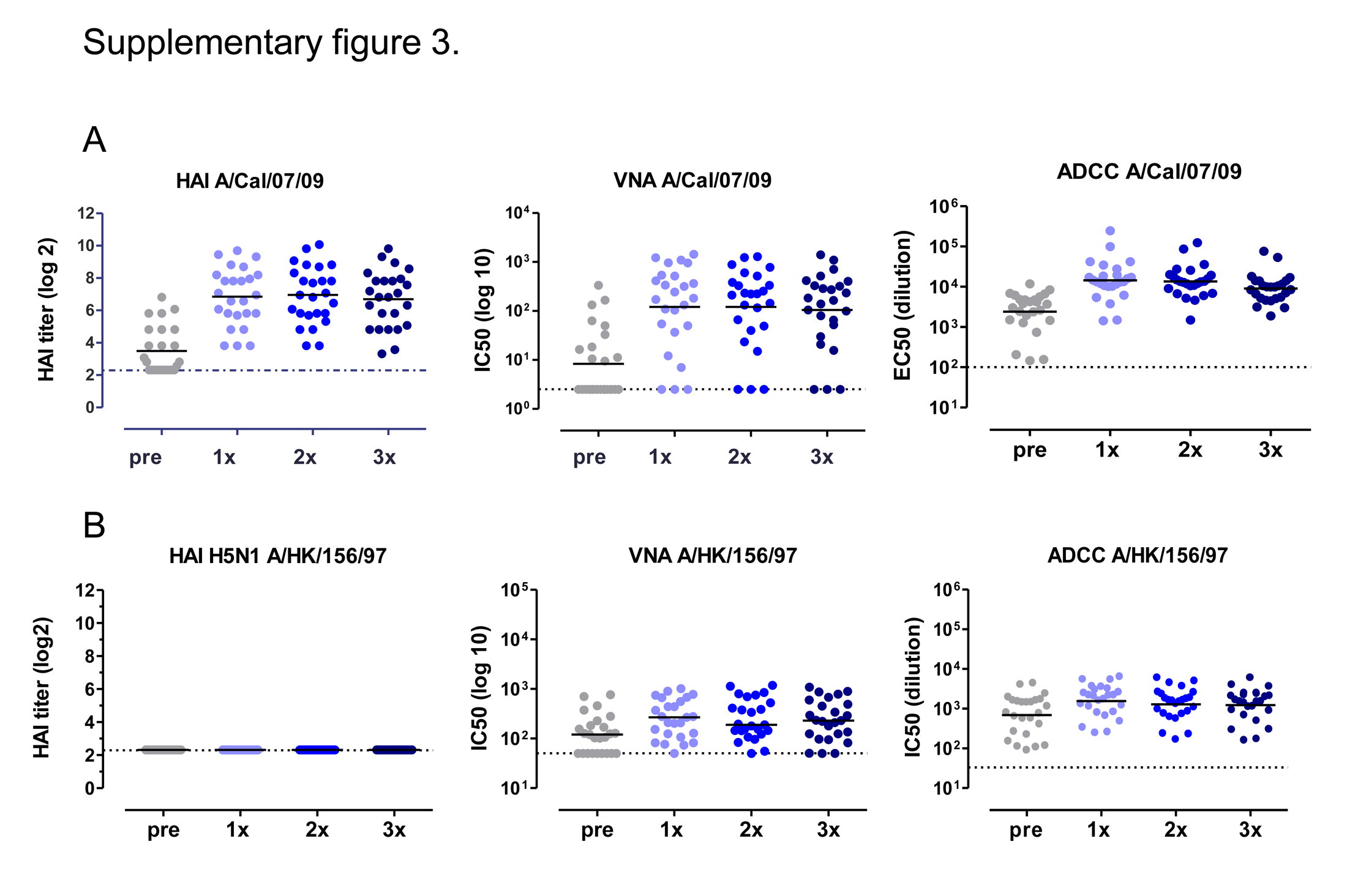

Supplement: Figure S3 — Virus challenge strain–specific HAI, VNA and ADCC titers remain constant after first immunization. HAI, VNA and ADCC titers against (A) H1N1 A/California/07/2009 and (B) H5N1 A/Hong Kong/156/97 are depicted for pre-vaccination serum and sera obtained after 1×, 2×, and 3× vaccinations. Dashed lines indicate background levels in the respective assays. The titers at all three post-vaccination visits are statistically significantly higher (p<0.001) than at the pre-vaccination visit for all assays except for HAI H5N1 where all titers fall below the detection limit. (TIF) [file pone.0103550.s003.tif]
